# Development of the PRECIOUS Short-Form (PRECIOUS-SF) quality of care measure for children with serious illnesses

**DOI:** 10.1186/s41687-025-00844-x

**Published:** 2025-01-24

**Authors:** Felicia Jia Ler Ang, Yin Bun Cheung, Mihir Gandhi, Rahul Malhotra, Truls Ostbye, Chetna Malhotra, Cristelle Chu-Tian Chow, Poh Heng Chong, Zubair Amin, Teresa Shu Zhen Tan, Eric Finkelstein

**Affiliations:** 1 Lien Centre for Palliative Care, Duke-NUS Medical School, Singapore, Singapore; 2 Programme in Health Services & Systems Research, Duke-NUS Medical School, Singapore, Singapore; 3 Tampere Center for Child, Adolescent, and Maternal Health Research: Global Health Group, Tampere University, Tampere, Finland; 4 Centre for Ageing Research and Education, Duke-NUS Medical School, Singapore, Singapore; 5 Duke Global Health Institute, Duke University, Durham, USA; 6 Children’s Complex and Home Care Services, KK Women’s & Children’s Hospital, Singapore, Singapore; 7 HCA Hospice Limited, Singapore, Singapore; 8 Paediatrics, National University of Singapore, Singapore, Singapore; 9 Khoo Teck Puat–National University Children’s Medical Institute, National University Hospital, Singapore, Singapore; 10 Department of Population Health Sciences, Duke University, Durham, USA

**Keywords:** Process assessment, Quality of care, Patient-reported measure, Process measure, Person-centered care, Psychometrics

## Abstract

**Background:**

Rising number of children with complex medical conditions necessitate regular healthcare quality evaluation to achieve optimal outcomes. To address the need for a periodic and quick assessment of quality of care in serious childhood illnesses, we developed a short version of previously validated 45-item PaRental Experience with care for Children with serIOUS illnesses (PRECIOUS) measure.

**Methodology:**

PRECIOUS was administered by parents of children living with serious illnesses at two time-points (baseline and two weeks) in an online survey. PRECIOUS Short-Form (PRECIOUS-SF) items were derived from the full PRECIOUS measure, which comprises five scales, using an exploratory factor analysis and best subset regression. The measurement properties of PRECIOUS-SF scales were assessed using the concurrent validity using Pearson correlation (*r*) with the PRECIOUS scales, internal consistency (Cronbach’s α) within each scale, convergent validity with overall QoC rating, and test-retest reliability (intraclass correlation coefficient, ICC) between baseline and two-week responses.

**Results:**

PRECIOUS-SF included 10 items across four scales – (1) access to financial and medical resources (2), collaborative and goal-concordant care (3), caregiver support and respectful care and (4) reduction of caregiving stressors. A fifth and optional scale was suggested for hospitalization-specific processes. PRECIOUS-SF scales correlated strongly with corresponding PRECIOUS scales (*r* = 0.91 to 0.98) and demonstrated satisfactory internal consistency (α = 0.77 to 0.91) and test-retest reliability (ICCs > 0.70).

**Conclusions:**

PRECIOUS-SF demonstrated internal consistency, convergent validity, test-retest reliability, and concurrent validity with PRECIOUS. PRECIOUS-SF offers a practical tool for routine quality of care assessment in pediatric serious illnesses for promoting timely service evaluation and quality improvement.

**Supplementary Information:**

The online version contains supplementary material available at 10.1186/s41687-025-00844-x.

## Abstract (plain language summary)

Regular evaluations of healthcare quality are essential for managing serious childhood illnesses. To address this, we developed and validated a short form of a comprehensive 45-item PaRental Experience with care for Children with serIOUS illnesses (PRECIOUS) measure. The short-form version of PRECIOUS (PRECIOUS-SF) includes four scales (access to financial and medical resources, collaborative and goal-concordant care, caregiver support and respectful care, and reduction in caregiving stressors). An optional hospitalization-specific processes scale can be included to assess hospitalization-related aspects of care. PRECIOUS-SF scales strongly correlate with the PRECIOUS measure scales, demonstrating its validity for assessing quality of care in pediatric serious illnesses. PRECIOUS-SF provides a practical and efficient method for routine quality of care assessment, facilitating timely implementation and quality improvement in clinical settings.

**Supplementary Information:**

The online version contains supplementary material available at 10.1186/s41687-025-00844-x.

## Introduction

The incidence of serious illnesses in children, including a spectrum of life-threatening and life-limiting conditions [1], has been increasing worldwide [2, 3]. These illnesses significantly affect the child’s quality of life and place a considerable burden on families and caregivers [4–6]. As medical advancements enable longer lifespans for these children, there is a growing demand for long-term care for many conditions such as severe cerebral palsy, congenital malformation syndromes or genetic syndromes, and cancers [7–9].

This evolving healthcare landscape underscores the importance of quality of care (QoC) measurements, which is recognized as a catalyst for improving health outcomes [10, 11]. QoC extends beyond treatment safety and effectiveness and includes the broader health and social care experience [10, 12]. Parents of seriously ill children navigate distinct health and social care challenges, assuming critical roles in managing their child’s complex medical, developmental, and emotional needs [4, 13, 14]. Thus, parent-reported experience measures (PaREMs) are crucial process measures of QoC [15–17]. In our prior work, we found that existing PaREMS mostly pertain to acute care or single healthcare setting [18]. There is a shortage of comprehensive QoC measures that are relevant over the serious illness trajectory and across varied healthcare settings, such as hospitals, community-based facilities, and home hospices.

We have previously developed and validated a QoC measure termed PaRental Experience with care for Children with serIOUS illnesses (PRECIOUS) to assess QoC for seriously ill children that can be used within and across healthcare sectors and over the serious illness trajectory [19]. Despite its merits, the original 45-item PRECIOUS measure may be too lengthy for routine and timely assessment in clinical settings, given the cognitive load and time required for completion. Therefore, our aim was to derive a short-form version of PRECIOUS (PRECIOUS-SF) and evaluate its measurement properties for validity, reliability, internal consistency, and comparability with the longer 45-item PRECIOUS measure.

## Methods

### Study design and participants

This prospective survey collected data from parents with seriously ill children at two points: baseline and a follow-up after two weeks. Children (< 18 years) were considered seriously ill if they had any of four conditions as defined by Together for Short Lives [1]: life-threatening conditions, conditions causing premature death, progressive conditions without cures, and severe conditions causing disability and health susceptibility. This broad inclusion criteria aimed to ensure the study’s findings were applicable across various primary diagnoses.

Parents were recruited from various organizations, including tertiary hospitals’ intensive care units and outpatient clinics, allied health services, charities, hospices, and parent support groups in Singapore. Bereaved parents, non-parent caregivers, and paid caregivers were excluded. Parents completed online surveys hosted on Qualtrics (Enterprise License) and received a cash voucher for participation. The baseline survey included sociodemographic questions, the 45-item PRECIOUS measure, the Quality of Children’s Palliative Care Instrument (QCPCI) [20], and Measure of Processes Of Care (MPOC-20) [16]. The follow-up survey at two weeks only included PRECIOUS to assess its test-retest reliability. In all measures, higher scores denote better QoC.

The study was approved by the National University of Singapore Institutional Review Board [NUS-IRB-2022-489].

### Measures

PRECIOUS is a comprehensive 45-item PaREM for assessing QoC, with each item having a 5-point Likert scale response score ranging from 0 (‘Never’) to 4 (‘Always’). The measure constitutes of five scales: (1) collaborative and goal-concordant care, (2) caregiver support and respectful care, (3) access to financial and medical resources, (4) reduction of caregiving stressors, and (5) hospitalization-specific processes. Each scale is scored as the average of its item scores, and ranged from 0 to 4, with a higher score indicating better QoC. Fifteen items have a ‘Not applicable’ response option to accommodate varying parental experiences like special needs schooling. The PRECIOUS also includes two screener questions to determine parental eligibility to respond to five items on recent hospitalization and home care. PRECIOUS was developed and validated through six sequential phases [18, 19, 21, 22]: (1) scoping review; (2) in-depth interviews; (3) expert panel review; (4) pre-testing (5), pilot-testing, and (6) assessment of measurement properties.

QCPCI evaluates hospital palliative care through 15 items scored on a 5-point Likert scale (0 to 4) and an additional 5-point overall QoC rating, resulting in four subscale scores and an overall QoC score. MPOC-20 assesses family-centeredness in community services with 20 items on a 7-point scale (1 to 7) for each item to generate five subscale scores. Both QCPCI and MPOC-20 are validated for QoC assessment in specific settings and intended for both acute and chronic care. Since these measures assess similar constructs, we found them suitable for evaluating the convergent validity of PRECIOUS, which was also designed to be applicable across settings.

### Derivation of candidate short-forms

To derive candidate short-forms for PRECIOUS-SF, we first leveraged the exploratory factor analysis previously conducted on the 45-item version of PRECIOUS [19]. Initially, we selected the top four items with the highest factor loadings from each scale to be considered for the short form (identified in Table 1). For the hospitalization-specific processes (HOSP) scale, which consists of only four items, we identified the top three items to avoid replicating the full scale. Previous studies suggest that a minimum of two to three items per scale will sufficiently represent the scale’s information [23, 24].

**Table 1:** Candidate items for PRECIOUS-SF identified from exploratory factor analysis of the 45-item PRECIOUS

Scale	Factor loading	Code	Abbreviated item description
Access to financial and medical resources	0.72	AR3	Access to sufficient financial support for non-medical expenses
	0.65	AR4	Allied health support for development goals
	0.64	AR1	Access to sufficient financial support for medical expenses
	0.57	AR2	Care worker/team that organized our child’s care across different care services
Collaborative and goal-concordant care	0.74	CC11	Responsiveness to medical issues
	0.71	CC5	Worked together towards common goals
	0.67	CC8	Informed about child’s condition
	0.66	CC7	Effort to build trust
Caregiver support and respectful care	0.83	SR6	Demonstration of care and concern
	0.83	SR8	Kind listening ear
	0.76	SR5	Listening to parental concerns
	0.73	SR4	Acknowledgment of parental effort
Reduction of caregiving stressors	0.88	RS8	Communication with school/day-care
	0.83	RS4	Support for family’s emotional needs
	0.81	RS6	Emotional support for child
	0.76	RS3	Information on specialized transport
Hospitalization-specific processes	0.94	HP2	Minimizing exposure to infectious diseases
	0.55	HP3	Opportunities for parent-child bonding
	0.53	HP1	Timely attention in Emergency Department

For each scale, we applied best subset regression to the baseline data to identify the best fitting candidate short-form models with different numbers of items (ranging from one to four items, except for the HOSP scale which varied from one to three items since the scale only contained four items in total), due to its focus on maximizing predictive accuracy and efficiently selecting the most informative items.

Best subset regression systematically evaluates all possible combinations of predictor variables to identify the subset of items which best fits the model. This method is distinguished by its exhaustive nature, examining all potential combinations, unlike stepwise regression methods which sequentially add or remove predictors [25]. While we considered alternative methods like confirmatory factor analysis (CFA) and item response theory (IRT) for their robust measurement insights, best subset regression was chosen to directly enhance the predictive validity of the short form. The best subset regression methodology has been successfully used in other studies for developing short-form measures, including for studies with a relatively smaller sample size [26, 27].

We used the Akaike Information Criterion (AIC) and Bayesian Information Criterion (BIC) to identify the best model among those with the same item counts, with preference given to models demonstrating lower (better) AIC and BIC values. AIC and BIC are mathematical methods used to evaluate and compare the goodness of fit and complexity of models, with lower values indicating a better model fit. If the AIC and BIC were not in agreement, we focused on the AIC as its emphasis on goodness-of-fit over simplicity better aligns with our objective of ensuring comparability between PRECIOUS-SF and PRECIOUS [28].

After identifying the best-fitting 1-, 2-, 3-, and 4-item (all but HOSP) models for each scale, we computed a score for each candidate PRECIOUS-SF scale by averaging relevant item scores.

### Evaluating measurement properties of candidate short-forms

Concurrent validity (correlation with the gold standard) of the PRECIOUS-SF scales with the corresponding scales from PRECIOUS was evaluated using Pearson’s correlation coefficient (*r*). Convergent validity (correlation between similar constructs) of PRECIOUS-SF scales with the overall QoC rating from QCPCI was assessed using Spearman’s correlation coefficient (*ρ).* Internal consistency among items of each PRECIOUS-SF scale was evaluated using Cronbach’s α. Finally, the test-retest reliability between baseline and follow-up PRECIOUS-SF scales was estimated using the intraclass correlation coefficient (ICC) [29]. We included parents who returned the follow-up survey within a month of the baseline, whose children were still alive at follow-up, and who reported no major change in their child’s status or care.

### Selecting among candidate short-forms

To select among the candidate short-forms for PRECIOUS-SF, we compared Pearson’s correlations between each candidate short-form scale and its corresponding scale from PRECIOUS. If the correlation (*r*) between the scales of the candidate PRECIOUS-SF and PRECIOUS did not exceed 0.9 (very strong relationship) [30], candidate short-forms with more items were considered. Furthermore, if the internal consistency (α) or test-retest reliability (ICC) of the candidate PRECIOUS-SF scales did not exceed 0.7, we considered candidate short-forms with more items [29, 31].

After selecting amongst candidate short-forms for each PRECIOUS-SF scale, we analyzed follow-up data to calculate Cronbach’s α for all selected short-forms and assessed their correlation with the scales of the 45-item PRECIOUS, to verify if the baseline findings were replicable.

### Conversion from PRECIOUS-SF scales to PRECIOUS scales

To predict each PRECIOUS scale using each corresponding PRECIOUS-SF scale, we used ordinary least squares regression with the PRECIOUS scale as the dependent variable and PRECIOUS-SF scale as the independent variable to develop a conversion formula for each scale separately.

## Results

### Sample description

A total of 152 parents completed the baseline survey. They had a mean (SD) age of 42.4 (7.3) years, with a majority being mothers (71%) of Chinese ethnicity (66.5%). Recruited parents’ children were distributed across all four Together for Short Lives serious illness categories [1, 32], with a slight majority being male (59.9%). The children’s ages ranged across all childhood developmental stages (infancy, early childhood, middle childhood and adolescence), with the mean duration of illness being 6.2 years. More details on participant characteristics are available in Supplementary Material Table 1.

### Derivation of candidate short-forms

Table 2 shows the best subset regression analysis results and correlation (*r)* between the candidate short-forms and PRECIOUS for scales on access to financial and medical resources (ACCR), collaborative and goal-concordant care (CGC), caregiver support and respectful care (SRC), and reduction of caregiving stressors (RCS). We present four best-fitting models for 1-, 2-, 3-, and 4-item models for each scale, except for hospitalization-specific processes (HOSP) scale, which had up to 3-item models.

**Table 2:** Results of best subset regression analysis and Pearson’s *r* between each candidate short-form model and the corresponding scale scores of the 45-item PRECIOUS measure

Scale (short form)	Number of items^a^	Items	Akaike Information Criterion (AIC)^b^	Bayesian Information Criterion (BIC)	Pearson’s *r* (with corresponding scale scores of the 45-item PRECIOUS measure)^c^
Access to financial and medical resources (ACCR-SF)	1	AR3	216.52	222.56	0.83
	2	AR2 AR3	77.13	86.20	0.93
	3	AR1 AR2 AR4	4.72	16.81	0.96
	4	AR1 AR2 AR3 AR4	-92.47	-77.35	0.98
Collaborative and goal-concordant care (CGC-SF)	1	CC8	120.60	126.65	0.85
	2	CC5 CC8	31.88	40.95	0.92
	3	CC5 CC7 CC11	-28.78	-16.68	0.95
	4	CC5 CC7 CC8 CC11	-57.48	-42.36	0.96
Caregiver support and respectful care (SRC-SF)	1	SR6	110.81	116.86	0.85
	2	SR6 SR8	49.30	58.37	0.91
	3	SR4 SR5 SR8	5.02	17.12	0.93
	4	SR4 SR5 SR6 SR8	-21.41	-6.29	0.94
Reduction of caregiving stressors (RCS-SF)	1	RS4	174.69	180.33	0.89
	2	RS4 RS6	107.36	115.82	0.94
	3	RS3 RS4 RS6	58.62	69.90	0.96
	4	RS3 RS4 RS6 RS7	13.85	27.95	0.97
Hospitalization-specific processes (HOSP-SF)^d^	1	HP3	93.22	98.01	0.80
	2	HP1 HP3	35.54	42.72	0.89
	3	HP1 HP2 HP3	-14.30	-4.72	0.95

^a^ Best-fitting models for 1-, 2-, 3-, and 4-item models per scale

^b^ Both AIC and BIC measure model fit while correcting for complexity, with BIC applying a stronger correction for parsimony; lower values indicate a better fit

^c^*r* > 0.9 indicates a very strong correlation between the short-form and full-scale measures

^d^ HOSP-SF models were limited to 1 to 3 items as the original scale had only 4 items, and using all would replicate the full scale

As Table 2 shows, AIC and BIC were better for short-forms with more items. However, for the ACCR, CGC, SRC, and RCS scales, two items were sufficient to achieve the target *r* > 0.9. The incremental gain in *r* by including more than two items was small.

### Measurement properties of candidate short-forms

Table 3 shows the results of measurement properties of the best 1-, 2-, 3- and 4-item short-form scales. For comparison, the results of each corresponding PRECIOUS scale are also presented. The difference in correlation with the overall QoC score from QCPCI between 1-item PRECIOUS-SF scales and corresponding PRECIOUS scales ranged from 0.04 (CGC) to 0.17 (ACCR). When two items were included per short-form scale, the differences narrowed to a range of 0.01 (CGC) to 0.08 (ACCR). Inclusion of additional items per scale did not consistently improve correlation with overall QoC. For example, further increasing to include three items per short-form scale narrowed this marginally to a range of 0 (ACCR, RCS) to 0.05 (SRC).

**Table 3:** Convergent validity, internal consistency and test-retest reliability of the candidate short form models

Scale	Number of items	Model	Spearman’s ρ (overall QoC)^a^	Cronbach’s α^b^	Intraclass correlation coefficient (ICC)^c^
Access to financial and medical resources (ACCR)	5	ACCR in 45-item PRECIOUS	0.38*	0.85	0.72
	1	AR3	0.21*	-	0.81
	2	AR2 AR3	0.30*	0.63	0.70
	3	AR1 AR2 AR4	0.38*	0.73	0.65
	4	AR1 AR2 AR3 AR4	0.35*	0.82	0.83
Collaborative and goal-concordant care (CGC)	12	CGC in 45-item PRECIOUS	0.46*	0.93	0.75
	1	CC8	0.42*	-	0.72
	2	CC5 CC8	0.45*	0.83	0.73
	3	CC5 CC7 CC11	0.45*	0.85	0.73
	4	CC5 CC7 CC8 CC11	0.46*	0.89	0.76
Caregiver support and respectful care (SRC)	15	SRC in 45-item PRECIOUS	0.50*	0.96	0.74
	1	SR6	0.45*	-	0.62
	2	SR6 SR8	0.45*	0.87	0.72
	3	SR4 SR5 SR8	0.45*	0.85	0.76
	4	SR4 SR5 SR6 SR8	0.46*	0.90	0.75
Reduction of caregiving stressors (RCS)	9	RCS in 45-item PRECIOUS	0.39*	0.95	0.86
	1	RS4	0.31*	-	0.82
	2	RS4 RS6	0.36*	0.87	0.86
	3	RS3 RS4 RS6	0.39*	0.90	0.87
	4	RS3 RS4 RS6 RS7	0.43*	0.89	0.88
Hospitalization-specific processes (HOSP)	4	HOSP in 45-item PRECIOUS	0.51*	0.74	0.78
	1	HP3	0.37*	-	0.56
	2	HP1 HP3	0.50*	0.50	0.66
	3	HP1 HP2 HP3	0.50*	0.68	0.66

^a^ Spearman’s *ρ*: Correlation between the short form and overall Quality of Care rating

^b^ Cronbach’s α: Measure of internal consistency, with values > 0.7 indicating acceptable reliability

^c^ Intraclass correlation coefficient (ICC): Test-retest reliability, reflecting stability of responses over time

* p < 0.05

For the short-form CGC, SRC and RCS scales, including just two items achieved internal consistency of α > 0.80. Short-form ACCR required three items to achieve α > 0.7, while including four items further improved α to 0.82. We were unable to attain α of 0.70 for the short-form HOSP scale, even when including up to three items.

Out of 152 parents who completed the baseline survey, 123 met the pre-defined criteria for inclusion in test-retest reliability analysis (returned follow-up survey within a month of baseline, children alive at follow-up, reported no major change in child’s status or care). The short-form ACCR, CGC, and RCS scales achieved ICC > 0.70 even with one item. Short-form SRC required two items to achieve ICC > 0.70. We were unable to attain ICC > 0.70 for the short-form HOSP scale, even when including up to three items.

### Proposed short-form

Based on the results, we propose a 10-item PRECIOUS-SF measure with four scales (Table 4): ACCR (4 items), CGC (2 items), SRC (2 items), RCS (2 items). The original ‘HOSP’ scale remains a 4-item standalone module that can be included according to the specific requirements of the user (i.e. team, service, or organization administering the measure).

**Table 4:** Descriptive summary of scales and items of the 10-item PRECIOUS-SF measure and add-on HOSP scale

Scale of the PRECIOUS-SF measure, and abbreviated items^a^	*N*	Mean	SD
Access to financial and medical resources (ACCR-SF) • Access to sufficient financial support for medical expenses (AR1) • Care worker/team that organized our child’s care across different care services (AR2) • Allied health support for developmental goals (AR4) • Access to sufficient financial support for non-medical expenses (AR3)	152	2.43	0.90
Collaborative and goal-concordant care (CGC-SF) • Worked together towards common goals (CC5) • Informed about child’s condition (CC8)	152	3.04	0.79
Caregiver support and respectful care (SRC-SF) • Demonstration of care and concern (SR6) • Provided a kind listening ear (SR8)	152	2.95	0.81
Reduction of caregiving stressors (RCS-SF) • Support for family’s emotional needs (RS4) • Emotional support for child (RS6)	129	1.98	1.26
Hospitalization-specific processes (HOSP) • Reasonable waiting time at Emergency Department (HP1) • Minimize exposure to infectious diseases (HP2) • Opportunities to bond with child in hospital (HP3) • Flexibility in caregivers at child’s bedside in Intensive Care (HP4)	81	2.90	0.70

^a^ Full measure is available at https://www.duke-nus.edu.sg/lcpc/resources/precious

#### Replicating the baseline findings using follow-up data

Next, we used data from follow-up survey to determine Cronbach’s α for all candidate short-forms and the correlation between these and the scales of the 45-item PRECIOUS measure, to verify if the baseline findings were replicable. Supplementary Material Table 2 shows these results, which are consistent with those from the baseline survey. The correlation between the four scale scores of the 10-item PRECIOUS-SF measure and the corresponding scale scores of the 45-item PRECIOUS measure was strong, ranging from 0.91 (CGC) to 0.98 (ACCR). Cronbach’s α was satisfactory for all scales, varying from 0.77 (ACCR) to 0.91 (RCS). We also noted that the ICCs of the proposed PRECIOUS-SF scales were similar to the original PRECIOUS scale ICCs.

### Conversion from PRECIOUS-SF to PRECIOUS scales

Table 5 presents the conversion equations derived from ordinary least squares regression for translating PRECIOUS-SF scales into their equivalent PRECIOUS scales. For example, the conversion equation for SRC is:

$${\text{SRC}}\,{\text{ = }}\,0.768\, + \,0.754*(SRC -SF)$$

**Table 5:** Conversion formulae between scales of the PRECIOUS and PRECIOUS-SF measures from ordinary least squares regression

Dependent variable^a^	Alpha (constant)	Beta coefficient	Independent variable^b^	*R* ^2^
ACCR	0.074	0.963	ACCR-SF	0.961
CGC	0.530	0.792	CGC-SF	0.849
SRC	0.768	0.754	SRC-SF	0.826
RCS	0.518	0.809	RCS-SF	0.882

Access to financial and medical resources (ACCR), Collaborative and goal-concordant care (CGC), Caregiver support and respectful care (SRC), and Reduction of caregiving stressors (RCS)

^a^ Scale of the PRECIOUS measure

^b^ Scale of the PRECIOUS-SF measure

To illustrate the conversion from PRECIOUS-SF to PRECIOUS scales, consider the following example: A parent completed the PRECIOUS-SF measure and had reported a score of 2.50 for SRC-SF scale. His/her score for SRC scale would be 0.768 + 0.754 (2.50) = 2.65.

For each PRECIOUS-SF scale, we also tested the inclusion of quadratic terms. However, these additions showed minimal improvement in the model’s accuracy. For instance, in the conversion from CGC-SF to the CGC scale, incorporating a quadratic term for the CGC-SF scale only increased the R^2^ value slightly, from 0.849 to 0.859 (Supplementary Material Table 3).

## Discussion

We have developed and conducted preliminary assessment of internal consistency, convergent validity, test-retest reliability and concurrent validity of a 10-item short-form ‘PRECIOUS-SF’ measure derived from the 45-item PRECIOUS measure. PRECIOUS-SF demonstrated satisfactory measurement properties and consistency with the long version. The PRECIOUS-SF, along with programming statements and a scoring algorithm, is hosted on the Duke-NUS Medical School website at https://www.duke-nus.edu.sg/lcpc/resources/precious. By facilitating service evaluation and enabling quality of care benchmarking, PRECIOUS-SF can support the development and evaluation of targeted interventions aimed at enhancing quality of care for seriously ill children. The conversion formula between PRECIOUS-SF scales and PRECIOUS scales may facilitate comparison and benchmarking across studies and/or sites that use different versions of the measure.

The PRECIOUS-SF measure offers a practical resource for both clinical settings and research. We recommend that stakeholders involved in quality of care assessments should also tailor their approach based on their specific goals. When the objective is to identify suboptimal performance at specific sites or identify families with unmet care priorities, initiating assessments with PRECIOUS-SF is a practical starting point. However, for example, if facilities or providers receive low scores on a specific scale of PRECIOUS-SF, they may opt to administer the comprehensive version of the scale from PRECIOUS. Alternatively, families who report low quality of care scores on certain scale(s) of PRECIOUS-SF may be re-assessed using the respective comprehensive PRECIOUS scale(s). This stepwise approach allows for a detailed analysis when needed, balancing between practicality and comprehensiveness. Meanwhile, the 45-item comprehensive PRECIOUS measure remains a valuable resource for in-depth research investigations, quality improvement initiatives, or new service evaluations.

The proposed PRECIOUS-SF measure has some inherited limitations from the PRECIOUS measure [19] and a few more. Firstly, while our study met the minimum recommended sample size for the exploratory factor analysis, a larger sample could have been ideal to minimize the noise and better separation of scales. We recommend further evaluation with a larger samples and other data-demanding methodologies (e.g., item response theory) to confirm the scale structure. Secondly, the short form was derived from the 45-item PRECIOUS measure, with the assumption that selected items perform consistently whether included in the full measure or used independently. This assumption is supported by previous studies indicating that quality of life assessments are typically unaffected by context effects [33, 34], but lacks evidence in the measurement of quality of care. Additional limitations of the PRECIOUS-SF measure also involve the 1-item model (AR3) having higher ICCs compared to the 2- and 3-item models of the ACCR-SF scale. As all inter-item correlations between AR1, AR2, AR3 and AR4 exceeded 0.4, it is unlikely due to low inter-item correlations. We posit that item AR3 may be understood and reported more consistently compared to items AR1and AR2, resulting in greater stability (reflected in a higher ICC) for the AR3-only model. Items AR1 and AR2, which evaluate *access to financial support for the child’s medical expenses* and the *organization of various care services*, may involve more subjective judgment. Although the underlying processes measured by these items may remain stable, their subjective nature might result in variability in parental interpretation over time. Thirdly, the ordinary least square regression model used for scale conversion may lack symmetry. However, in many practical applications, the slight lack of symmetry in may not significantly impact the overall utility of the short form. A study with a larger sample size should be conducted in the future for a more complex and accurate mapping algorithm. Lastly, the measurement properties of PRECIOUS-SF were evaluated using the same study sample used for evaluating the measurement properties of PRECIOUS. Hence, the generalizability of the findings could be limited. A future study in diverse populations and/or datasets will be helpful in better understanding the performance of the measure in different settings.

### Conclusion

We developed a 10-item short-form of the PRECIOUS measure, demonstrating acceptable internal consistency, convergent validity, test-retest reliability, and its comparability with the longer 45-item PRECIOUS measure. The PRECIOUS-SF measure has the potential to serve as a reliable, valid, and practical tool for more rapid and routine quality of care assessment in pediatric serious illnesses. Although the comprehensive PRECIOUS measure provides detailed insights into care processes, the flexibility to choose between PRECIOUS and short-form of PRECIOUS (PRECIOUS-SF) empowers stakeholders to select the most suitable version for their specific needs. This dynamic approach not only enhances quality of care assessments but may promote efficient and effective use of either and/or both measures in diverse healthcare contexts.

## Electronic supplementary material

Below is the link to the electronic supplementary material.

Supplementary Material 1

Supplementary Material 2

Supplementary Material 3

## Data Availability

Mplus scripts and Stata do. files for the full PRECIOUS measure are available on Open Science Framework, while de-identified raw data is available upon request with an approved IRB.
