# Supplementary material for: Development of the PRECIOUS Short-Form (PRECIOUS-SF) quality of care measure for children with serious illnesses
Source: J Patient Rep Outcomes. 2025 Jan 24;9:12. doi: 10.1186/s41687-025-00844-x (PMC11759730; doi:10.1186/s41687-025-00844-x)
Supplement: Supplementary file 1 — Supplementary Material 1 [file 41687_2025_844_MOESM1_ESM.pdf]

Supplementary Material Table 1. Parent and child participant characteristics (N = 152) in the 45-item PRECIOUS validation study

Characteristics		Mean (SD) or N (%) *	
**Mean Age in years of Parent (SD)**		42.4	7.3
**Gender**			
	Female	108	71.0
	Male	44	29.0
**Ethnicity**			
	Chinese	101	66.5
	Malay	38	25.0
	Indian	7	4.6
	Others	6	4.0
**Highest education**			
	Primary education or less	4	2.6
	Secondary or ITE †	27	17.8
	Post-secondary	121	79.6
**Caregiving roles (Answered 'Yes')**			
	Make decisions about treatments the child receives	150	98.7
	Physically provide care to child (e.g., help with day-to-day activities)	138	90.8
	Pay for the medical and health care expenses	137	90.1
	Ensure provision of care (e.g., supervising a paid caregiver to look after child)	100	65.8
**Mean hours spent caregiving per week (SD)**		92.5	55.9
**Employment status**			
	Full-time employment	80	52.6
	No current employment	59	38.8
	Part-time employment	9	5.9
	Others	4	2.6
**Marital status**			
	Married	145	95.4
	Divorced/separated	5	3.3
	Never married	2	1.3
**Respondent status**			
	Myself only	143	94.1
	Myself, with assistance from a caregiver who is not the child's parent	6	4.0
	Myself, with assistance from another parent	2	1.3
	Myself, with assistance from a non-caregiver	1	0.7
**Child's illness category‡**			
	Category 1	50	32.9
	Category 2	25	16.4
	Category 3	24	15.8
	Category 4	53	34.9

**Child's gender**			
	Female	61	40.1
	Male	91	59.9
**Child's ethnicity**			
	Chinese	98	64.5
	Malay	38	25.0
	Indian	8	5.3
	Others	8	5.3
**Child's age group**			
	0 – 1 years (Infants)	11	7.6
	1 – 5 years (Early Childhood)	40	27.8
	6 – 12 years (Middle Childhood)	45	31.3
	13 – 18 years (Adolescence)	56	38.9
**Mean duration of Child's illness in Years (SD)**		6.2	4.9

*Mean and standard deviation (SD) for continuous variables; frequency (N) and percent for categorical variables

† Institute of Technical Education (ITE) is a government-owned institution providing vocational and industrial training

‡ Categorization as defined by Together for Short Lives, United Kingdom: Category 1. Life-threatening conditions for which curative treatment may be feasible but can fail; Category 2. Conditions where premature death is inevitable; Category 3. Progressive conditions without curative treatment options; Category 4. Irreversible but non-progressive conditions causing severe disability, leading to susceptibility to health
