# Supplementary material for: Development of the PRECIOUS Short-Form (PRECIOUS-SF) quality of care measure for children with serious illnesses
Source: J Patient Rep Outcomes. 2025 Jan 24;9:12. doi: 10.1186/s41687-025-00844-x (PMC11759730; doi:10.1186/s41687-025-00844-x)
Supplement: Supplementary file 2 — Supplementary Material 2 [file 41687_2025_844_MOESM2_ESM.pdf]

Supplementary Material Table 2. Measurement properties of scales in the follow-up survey

Number of items	Short-form (SF) Scale [a]	*r* (with scale scores of the 45-item PRECIOUS measure)	Cronbach's α
	**ACCR-SF**		
4	AR1 AR2 AR3 AR4	0.98	0.77
	**CGC-SF**		
2	CC5 CC8	0.91	0.78
	**SRC-SF**		
2	SR6 SR8	0.92	0.87
	**RCS-SF**		
2	RS4 RS6	0.95	0.91
[a] Access to financial and medical resources (ACCR), Collaborative and goal-concordant care (CGC), Caregiver support and respectful care (SRC), and Reduction of caregiving stressors (RCS).			
