# Supplementary material for: Development of the PRECIOUS Short-Form (PRECIOUS-SF) quality of care measure for children with serious illnesses
Source: J Patient Rep Outcomes. 2025 Jan 24;9:12. doi: 10.1186/s41687-025-00844-x (PMC11759730; doi:10.1186/s41687-025-00844-x)
Supplement: Supplementary file 3 — Supplementary Material 3 [file 41687_2025_844_MOESM3_ESM.pdf]

Supplementary Material Table 3. Conversion formulae from ordinary least squares regression including quadratic terms.

Dependent variable [a]	Alpha (constant)	Beta coefficient(s)	Independent variable (s) [b]	R^2
ACCR	0.074	0.963	ACCR-SF	0.961
ACCR	0.129	0.908<br>0.012	ACCR-SF<br>ACCR-SF^2	0.961
CGC	0.530	0.792	CGC-SF	0.849
CGC	1.01	0.400<br>0.072	CGC-SF<br>CGC-SF^2	0.859
SRC	0.768	0.754	SRC-SF	0.826
SRC	1.16	0.437<br>0.058	SRC-SF<br>SRC-SF^2	0.832
RCS	0.518	0.809	RCS-SF	0.882
RCS	0.628	0.616<br>0.050	RCS-SF<br>RCS-SF^2	0.887

[a] Access to financial and medical resources (ACCR), Collaborative and goal-concordant care (CGC), Caregiver support and respectful care (SRC), and Reduction of caregiving stressors (RCS).
[b] Short-form (SF)
